# Interactive Internet-Based Motivational Interviewing Training for HIV Counseling Support Staff to Improve Health Communication in HIV Care Interactions: Protocol for Training Development and a Pilot Randomized Controlled Trial

**DOI:** 10.2196/82241

**Published:** 2026-01-07

**Authors:** Iván C Balán, Onna R Brewer, Bryan A Kutner, Rebecca Giguere

**Affiliations:** 1 Center for Translational Behavioral Science, Department of Behavioral Science and Social Medicine College of Medicine Florida State University Tallahassee, FL United States; 2 Division of General Internal Medicine Department of Psychiatry and Behavioral Sciences Albert Einstein College of Medicine Bronx, NY United States

**Keywords:** HIV, implementation science, internet-based, Motivational Interviewing, training

## Abstract

**Background:**

HIV counseling support staff (CSS) play a crucial role in HIV care outcomes, providing essential access to HIV test counseling, linkage-to-care support, adherence counseling, peer support, and navigation. Effective training in evidence-based interventions like motivational interviewing (MI) is imperative to maximize the impact of CSS in enhancing HIV care outcomes. MI is a collaborative, goal-oriented communication method aimed at bolstering an individual’s motivation and movement toward specific goals by eliciting and exploring personal arguments for change. MI has demonstrated efficacy across various medical and mental health outcomes, including its significant impact on the status-neutral HIV Care Continuum.

**Objective:**

The objective of this study is to develop and pilot an interactive internet-based MI training program for HIV counseling support staff (iMI4HIV), aiming to enhance their communication skills in HIV care interactions.

**Methods:**

The iMI4HIV Study will have 2 phases. In phase 1, Development, we will conduct formative focus groups (FGs) with CSS (n=16) to identify HIV care interactions for demonstration videos and to assess acceptability of gamification components; create MI demonstration videos depicting key interactions from status-neutral HIV Care Continuum; program iMI4HIV (including skills development tasks, gamification components, etc); and pilot a virtual MI (VMI) workshop followed by iMI4HIV (n=8). We will also conduct qualitative interviews with 8 leaders from HIV care agencies to explore their use of existing online MI training programs, acceptability of iMI4HIV, and possible facilitators and impediments to its adoption for training HIV CSS. In phase 2, pilot randomized controlled trial (RCT), we will randomize 30 CSS at a 2:1 ratio (VMI workshop + iMI4HIV vs VMI workshop + waitlist control) to assess the feasibility and acceptability of iMI4HIV and to pilot the RCT processes and assessments for a future efficacy trial. We will also explore the preliminary impact of iMI4HIV on MI skills acquisition.

**Results:**

The CSS focus groups began in August 2024, and programming of the training is near completion. We plan to conduct a pilot with 8 participants by the end of 2025, to obtain feedback and conclude phase 1 of the study. The phase 2 pilot RCT is expected to be launched in February 2026, with all data collection to be completed by October 2026.

**Conclusions:**

Our pilot study aims to demonstrate the feasibility and acceptability of an internet-based MI training program for HIV CSS. If successful, this training program has the potential to enhance the delivery of evidence-based interventions in HIV care settings, ultimately improving patient outcomes and adherence to treatment protocols. Further research and larger trials will be needed to confirm these findings and refine the training approach.

**International Registered Report Identifier (IRRID):**

DERR1-10.2196/82241

## Introduction

### Background

HIV counseling support staff (CSS) provide key services for HIV care outcomes, such as HIV test counseling, linkage to care, adherence counseling, peer support, and care navigation. Interventions delivered by CSS have increased HIV prevention [1,2], testing [3], linkage to care [4,5], treatment engagement [6], and adherence [4-7]. Nevertheless, to maximize patient outcomes, community-based CSS could benefit greatly from training to ensure competent delivery of evidence-based interventions, including motivational interviewing (MI).

MI is “a collaborative, goal-oriented method of communication aimed at strengthening an individual’s motivation for and movement toward a specific goal by eliciting and exploring the person’s own arguments for change” [8]. MI has proven efficacy in improving substance use [9,10], mental health [11,12], medical [13-17], and other outcomes across the HIV Care Continuum (HCC) in adults and adolescents [3,6,18-36]. MI has also been shown to improve linkage to and retention in care as well as treatment adherence [8-16], including among adolescents [6,26,31]. MI is particularly useful as an intervention because it requires CSS to master one set of skills applicable across the HCC instead of a variety of interventions for each step of the HCC. Furthermore, because it is efficacious even during brief interactions [37], MI can be integrated into all aspects of work typically conducted by CSS in HIV care settings. MI is embedded in HIV care guidelines, including those of the US Department of Health and Human Services and the New York State Department of Health [32,34], and training in MI is often offered through the national network of regional AIDS Education and Training Centers (AETCs).

However, mastering MI is not easy [38-43]. MI entails adhering to standard counseling tools (ie, open-ended questions, affirmations, reflections, and summaries) with an empathic, client-centered, collaborative approach. MI also includes a more difficult, technical component—the systematic elicitation and reinforcement of change talk (statements made by clients that argue for change, eg, “I need to get better at taking my pills”) and avoiding evocation of sustain talk (statements made by clients that argue for not changing, eg, “It’s hard to remember taking my pills”). Research on MI’s mechanisms of action has shown that more change talk and less sustain talk during a session are related to better outcomes [44-46]. Also, counselor behaviors such as advising, confronting, directing, and warning clients are associated with greater sustain talk, whereas counselor behaviors such as affirming strengths or effort, emphasizing client autonomy, and being supportive are associated with increased change talk [47,48]. Thus, to facilitate behavior change, the goal of an MI counselor is to interact with the client in a manner that facilitates change talk and inhibits sustain talk [38,48].

Standard MI training often consists of a 2-day workshop (about 14 hours total) with didactic presentations and many practice and experiential exercises. After this, attendees typically demonstrate an increase in MI relational skills (eg, asking permission before sharing information and more collaborative interactions). Demonstration of MI technical skills, such as using more open-ended questions and reflections, especially complex reflections [49-52] that are key components of MI’s mechanisms of action, is less likely after a 2-day workshop. Furthermore, acquired skills tend to diminish over the following 8-12 weeks without postworkshop feedback and coaching [53-55].

Studies have shown that CSS can become competent in MI [6,56], but in real-world settings, the training needed for CSS to attain MI competency is not readily available. This is a key challenge to disseminating and implementing MI [38,42,43]. Thus, we need to develop novel training approaches that are effective at teaching the technical MI skills that are key to MI’s efficacy and make these trainings more easily accessible to CSS in community settings. To ensure that MI retains its demonstrated efficacy in improving HCC outcomes, these MI training approaches need to be scalable so that they can both reach CSS working in HIV care settings and effectively increase their MI skills.

The World Health Organization has called for digital training for health care providers [57] and the broad use of online training for other behavioral interventions [58-60]. However, the use of technology in MI training has remained mostly limited to video demonstrations and brief didactic webinars that describe MI and provide examples of MI skills—even among the many MI-based health interventions that have been developed [61-63]. Findings from studies of technology-driven MI training have been encouraging. For example, multisession synchronous online MI training is feasible and effective [63,64], with no difference in counselor skill acquisition between in-person and online workshops [64]. Mitchell et al [65] used Second Life, a virtual world, to train physicians to increase acceptance of colorectal screening using 3 sessions of combined prerecorded MI tutorials, two 90-minute group role-play and feedback sessions with an MI coach, and between session practices, and thereby increased physician’s MI global skills related to client-centeredness (there was no assessment of individual technical skills). Other projects have begun to use avatars and simulated patient interactions to build MI skills [66-68]. These allow users to interact with virtual patients who are trained to respond in a certain manner to the words being used by the provider. To date, however, there are no studies that report on the efficacy of these training approaches, whether they are best used for beginner or advanced learners, or whether they need to be combined with other training approaches. Also, there are significant concerns about the costs to develop these patient interactions, especially because each simulation has to be constructed individually using words that are typical in the subject area the training addresses [68]. Finally, although technology-based MI training has high acceptability among users [63-68] and has shown preliminary evidence of efficacy in building MI skills [64,65], there are challenges to participant engagement and retention [63,68,69], highlighting the need for further research on novel technology-based MI training approaches to improve user engagement.

This study seeks to address a critical need for novel training approaches to build skills among CSS to deliver MI in health care interactions across the status-neutral HCC. Referred to as “iMI4HIV” (internet-based motivational interviewing training program for HIV counseling support staff), this study will offer a new approach to online MI training, specifically for HIV care settings. First, it will offer comprehensive interactive online training (10-12 hours) aimed at helping users learn how to use MI across a range of HCC interactions. Second, it will approach asynchronous online MI training in a completely novel way, using multiple MI video demonstrations combined with gamified exercises to sequentially build skills that are key to MI’s mechanisms of action, specifically the relational and technical skills essential for increasing change talk and inhibiting sustain talk. Third, it will integrate a variety of gamification and skills training components to increase engagement and appeal across a range of users [70-84]. Finally, this study includes a detailed framework for developing and evaluating the iMI4HIV strategy that includes HCC stakeholders, MI subject matter experts, and HCC learners in the development and piloting phases of the study, using mixed methods to evaluate the learner experience, engagement, and outcomes for MI learning.

### Study Objectives

The overall goal of the study is to develop and pilot an interactive, gamified, online training program in MI for HIV CSS to improve health communication in status-neutral HIV care interactions. This National Institutes of Health (NIH) R34 (Clinical Trial Planning Grant) study will allow us to pilot all the components and build the necessary research infrastructure for a subsequent randomized controlled trial (RCT) that is fully powered to assess the efficacy of iMI4HIV in increasing MI skills among CSS in HIV care interactions. The specific aims of this R34 study are as follows:

- Develop iMI4HIV, an interactive, online, gamified MI training program specifically tailored toward CSS in HIV care settings to improve their MI skills with HIV care clients.
- Conduct a pilot RCT of a virtual motivational interviewing (VMI) workshop + iMI4HIV versus VMI workshop + waitlist control (n=30) to (1) assess the feasibility and acceptability of iMI4HIV as measured by participant completion rate and retrospective acceptability ratings of iMI4HIV, and (2) explore preliminary findings on the effects of iMI4HIV on MI skills acquisition using Standard Patient Interactions (SPIs).
- Explore experiences (including engagement, obstacles, and facilitators) of completing iMI4HIV via in-depth interviews with pilot study participants.

In this paper, we present the proposed procedures for developing iMI4HIV, conducting the pilot RCT, and assessing its acceptability among HIV CSS.

## Methods

### Study Design

#### Overview

This study will have 2 phases. In phase 1 (Development), we will conduct formative focus groups (FGs) with CSS (n=16) to identify HIV care interactions for iMI4HIV videos and to assess the acceptability of gamification components; create MI demonstration videos depicting key interactions from status-neutral HCC and program iMI4HIV (including skills development tasks, gamification components, etc); and pilot the VMI workshop and iMI4HIV (n=8). We will also conduct qualitative interviews (QIs) with 8 leaders from HIV care agencies to explore their use of existing online MI training programs, acceptability of iMI4HIV, and possible facilitators and impediments to its adoption for training HIV CSS. In phase 2 (Pilot RCT), we will randomize 30 CSS at a 2:1 ratio (VMI workshop + iMI4HIV vs VMI workshop + waitlist control) to assess the feasibility and acceptability of iMI4HIV and to pilot the RCT processes and assessments for a future efficacy trial. We will also explore preliminary findings on the impact of iMI4HIV on MI skills acquisition. At the outset of the study, we will convene a Community Advisory Board (CAB) composed of 7 members, including individuals involved with HIV-related community health worker (CHW) training programs and HIV CSS with at least 1 year of experience providing services in HIV care settings. The CAB will meet via videoconferencing quarterly throughout the study.

#### Recruitment of CSS

Recruitment of participants will occur through 2 main recruitment streams. First, we will work with our partners at the Northeast and Caribbean AIDS Education and Training Center (NECA AETC) to help us recruit CSS through their network of collaborating HIV care agencies (a maximum of 2 CSS per clinic). Second, we will recruit through HIV-focused CHW training programs. These 2 recruitment streams will provide us with a group of CSS trainees that will be diverse in terms of HIV counseling experience and training. Eligibility criteria for all CSS study participants will be (1) aged 18 years or older, (2) currently works in a CSS role to improve HIV care outcomes, (3) English-language fluency (since iMI4HIV will only be developed in English during this study), and (4) access to a laptop, desktop, or tablet computer to attend the virtual training and complete iMI4HIV.

### Phase 1: Development

#### MI Training Content Development

We envision approximately 10-12 hours of training content organized by HCC component (Textbox 1). For each component, videos (each 8-18 minutes in duration) will be developed, highlighting different HCC-related interactions. The videos, by themselves, will serve as a resource to demonstrate proficient MI practice in these interactions. Furthermore, we will incorporate game-like interactive exercises aimed at building key MI skills that are critical to triggering its mechanisms of action, such as differentiating between open and closed questions, simple and complex reflections, and identifying and responding to change talk and sustain talk. In iMI4HIV, we will include some of the most frequently used gamification components (eg, points, feedback, levels, and badges of recognition) [75-78] while eschewing others, such as leaderboards (sharing a user’s points with all users to create competition), which may be counterproductive for this type of learning intervention [84]. The gamification components likely to be embedded in iMI4HIV include points to award learners for correct responses, feedback to encourage or prevent specific activities in order to improve performance, levels to stipulate that only users who attain a certain achievement can progress to the next level, and badges as a symbolic representation of a user’s achievement or competence.

**Components of an internet-based motivational interviewing training program for HIV counseling support staff.**

**Video content**

- Motivational interviewing (MI) basics
  - MI spirit
  - Four tasks of MI
  - MI micro skills: open-ended questions, affirmations, reflections, and summaries
  - Evoking, recognizing, and reinforcing change talk
  - Responding to sustain talk
  - Planning for change
- HIV testing
  - Overcoming ambivalence to testing
  - Facilitating regular testing
  - Reducing risk behaviors
- Linkage to care
  - Overcoming ambivalence to entry to care after HIV-positive results
  - Linkage to pre-exposure prophylaxis or other prevention services
  - Building motivation for linkage to care to ancillary services (mental health and substance abuse treatment)
- Retention in care
  - Exploration of the patient’s perceived benefits of staying in care
  - Collaborative planning to overcome obstacles to retention in care
  - Overcoming ambivalence to entry into care after HIV-positive results
  - Linkage to pre-exposure prophylaxis or other prevention services
  - Building motivation for linkage to care to ancillary services (ie, mental health and substance abuse treatment)
- Treatment adherence and viral suppression
  - Exploring the client’s views on adherence
  - Building motivation for adherence
  - Using viral load and CD4 lymphocyte counts to build motivation to adhere

**Sample tasks**

- Self-reflection exercises
- Brief didactic presentations
- Animation
- Gamification (points for picking open-ended questions or choosing the best reflection; clicking when you hear change talk)
- Coding brief segments for MI fidelity

Development of iMI4HIV will be guided by the User Centered Rapid Application Development process (UCRAD) [85,86]. UCRAD merges the streamlined, iterative Rapid Application Development (RAD) approach [87] with the User Centered Design approach [88,89], which engages intended users throughout the development process. This integrated approach aims to develop successful computer apps with good functionality, simple features, and a usable interface [90]. As such, intended users are given access to prototypes of the program, allowing them to provide feedback before the next iteration. We expect the process to take approximately 12 months in this study to produce the many scenarios and components of iMI4HIV. UCRAD uses a 3-stage process:

#### Predesign and Interface Prototyping

To accomplish this stage, we will obtain feedback from the CAB regarding the preliminary plans for iMI4HIV, FGs with CSS, and QIs with HIV care agency leaders:

- FG processes. We will conduct 2 real-time, 90-minute synchronous online FGs, each with 8 CSS, using a HIPAA (Health Insurance Portability and Accountability Act)-compliant video conference platform that allows for quantitative polling of participants regarding key attributes of iMI4HIV. The FGs will elicit individual- and group-level perspectives on topics that include (1) challenges faced in helping patients engage in care and obtain or sustain viral suppression, (2) prior MI training and use with patients, (3) challenges to learning and delivering MI, (4) feedback on iMI4HIV and different gamification components (eg, accumulating certain number of points before being able to proceed to next stage; preferences for types of games), and (5) other recommended training content. To obtain feedback on the gamification components, simple gamification components will be integrated into the FG discussion so that participants can provide feedback based on the gamification experience.
- QIs with HIV Care Agency Leadership. We will interview 8 leaders (including program directors who directly oversee CSS) from HIV care agencies associated with the NECA AETC. Given the extensive existing literature on barriers and facilitators to MI implementation, these interviews will focus more specifically on (1) knowledge and use of internet-based MI training, (2) barriers and facilitators to internet-based MI training they are currently using, (3) presentation and feedback on iMI4HIV, (4) recommendations for additional content, and (5) barriers and facilitators to CSS completing iMI4HIV.
- Rapid data analysis. Within 48 hours of completing each FG and QI, we will initiate a rapid data analysis [91,92] using a qualitative matrix [93,94] onto which participant responses will be entered to quickly organize and summarize FG findings. Summarized findings will be shared with the CAB and the external MI training consultants to obtain their feedback.

#### System Architecture and Coding

We will convene an iMI4HIV development team composed of MI trainers, program developers, and 4 CSS (selected from FG participants for their contributions and comfort in providing feedback). This team will meet monthly online over 6 months. Prior to each meeting, CSS will be asked to use prototype versions of a different iMI4HIV module (including the video, MI learning tasks, and gamification) and provide feedback. During the meeting, we will explore their experience completing the module, including what they liked or disliked, their reactions to the learning tasks and gamification components, and their overall experience of interacting with iMI4HIV.

#### Deployment

We will recruit 8 CSS from HIV care agencies and CHW training programs to pilot all training components. They will complete a 15-hour (3 days, 5 hours per day) HIV-focused VMI workshop conducted by experienced MI trainers that will consist of an overview of MI and extensive opportunities for joint interactive and experiential exercises conducted as a group and in dyads in different virtual rooms.

After completing the VMI workshop, participants will be sent an online questionnaire to provide feedback on the workshop and will be given access to iMI4HIV, which they will be asked to complete within 4 weeks. After completing iMI4HIV, or 5 weeks after being provided access, they (including those who were assigned to but did not complete iMI4HIV) will be asked to complete (1) an online questionnaire to assess acceptability of iMI4HIV and (2) an audio-recorded qualitative telephone interview to assess (a) overall acceptability of training program (workshop and iMI4HIV), (b) likes and dislikes of the program, (c) experience with gamification components, (d) difficulties encountered, and (e) feedback for the final version of the training program.

### Phase 2: Pilot RCT

#### Overview

The overall aim of phase 2 is to assess the feasibility and acceptability of iMI4HIV and prepare the necessary research infrastructure and processes for a future fully powered RCT to test its efficacy in increasing MI skills among CSS in HIV-related interactions. Thus, we will recruit 30 CSS and randomize them using a 2:1 ratio (VMI workshop + iMI4HIV vs VMI workshop + waitlist control).

**Figure 1:**
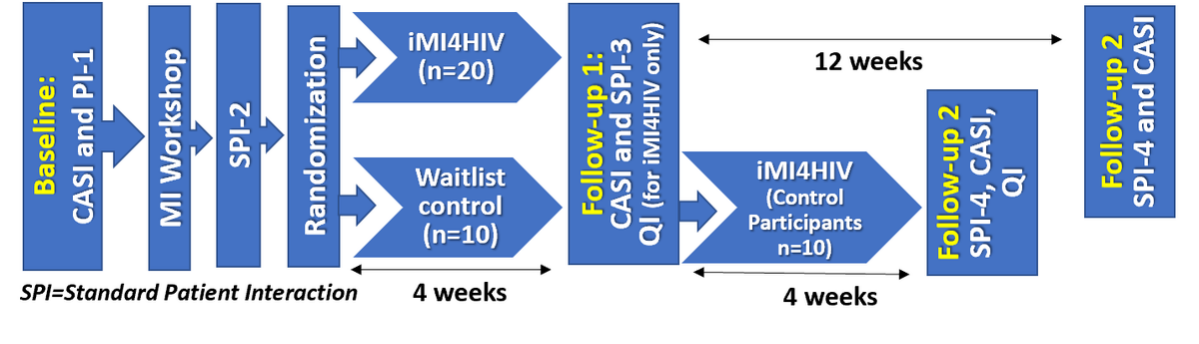
Phase 2 pilot randomized controlled trial overview. CASI: computer-assisted self-interview; iMI4HIV: internet-based Motivational Interviewing training program for HIV CSS; MI: Motivational Interviewing; QI: qualitative interview.

Figure 1 presents an overview of the phase 2 RCT procedures, detailing an RCT with a waitlist control arm which will gain access to iMI4HIV after completion of the Follow-Up 1 assessment. This design allows us to achieve the specific aims of the study as well as gain initial insights into the effects of iMI4HIV on MI skills and how these skills may be retained after 3 months. After all participants complete a baseline computer-assisted self-interview (CASI), they will complete SPI-1, then take part in the VMI workshop. After that, they complete SPI-2 and are then randomized into iMI4HIV versus a waitlist control. After 4 weeks, all participants complete Follow-Up 1, which consists of SPI-3, a follow-up CASI, and for only those in the iMI4HIV arm a qualitative interview. This will allow us to assess the feasibility and acceptability of iMI4HIV among those who were randomized into that arm (n=20). At this assessment point, we will also explore the effects of iMI4HIV on MI skills by comparing MI fidelity ratings of the SPI-3 between the intervention and control groups.

After Follow-Up 1, those in the control arm will be given access to iMI4HIV; after 4 weeks, only this group will complete Follow-Up 2, which will consist of SPI-4, a follow-up CASI, and an in-depth interview, which will provide additional acceptability data. Participants originally randomized into iMI4HIV will undergo their Follow-Up 2 after 12 weeks (SPI-4 and CASI, no QI). This will allow us to gain insights into retention of MI skills after 3 months, a key data point for a subsequent efficacy trial of iMI4HIV in which we would want to assess maintenance of MI skills. Community-based organizations rarely offer MI coaching to staff after a training workshop. Thus, we will not offer MI coaching in either study arm to focus on assessing the preliminary effects of iMI4HIV on participants’ MI skills.

#### Recruitment of CSS

We will work with HIV care clinics associated with the NECA AETC and CHW training program partners to enroll 30 CSS in the RCT. Interested CSS will be instructed to contact the study research assistant who will screen them for eligibility. Eligibility criteria will be as described above for the phase 1 CSS participants. If these criteria are met, research staff will send them an “enrollment email,” which will include a link to an online informed consent form.

Our assessment of acceptability of iMI4HIV will be guided by the Theoretical Framework of Acceptability [95] (TFA; refer to components in Textbox 2), which was derived from a review of the literature on acceptability, and views acceptability as “a multi-faceted construct that reflects the extent to which people delivering or receiving a healthcare intervention consider it to be appropriate, based on anticipated or experienced cognitive and emotional responses to the intervention” [95]. The TFA has guided qualitative and mixed methods research on the acceptability of health care interventions among patients and providers [96-99]. The TFA’s 7 domains have informed the development of a generic TFA quantitative assessment with Likert-type items to assess each component of the model [100].

**Components of the Theoretical Framework of Acceptability.**

**Theoretical Framework of Acceptability construct definitions**

• Affective attitude: how the individual feels about the intervention

• Burden: perceived amount of effort that is required to participate in the intervention

• Ethicality: the extent to which the intervention has a good fit with an individual’s value system

• Intervention coherence: the extent to which the participant understands the intervention and how it works

• Opportunity costs: the extent to which benefits, profits, or values are given up to engage in the intervention

• Perceived effectiveness: the extent to which the intervention is perceived as likely to achieve its purpose

• Self-efficacy: the participant’s confidence that they can perform the behaviors required to participate in the intervention

#### Baseline Assessments

After enrolling, participants will receive an email with a link to the CASI to be completed within 7 days and will be scheduled for their SPI-Baseline session, which will be audio-recorded and rated for MI fidelity. The CASI and the SPI-Baseline must be completed prior to the MI workshop. The baseline CASI assessment will include the following:

- Demographics. This will capture information on age, gender, race, ethnicity, education, primary language, counseling training, and prior MI training.
- MI Knowledge and Attitudes Test (MIKAT) [101], a 29-item assessment that has shown sensitivity to detect change in MI consistent and inconsistent behavior in pre- and posttraining comparisons [101-104], with acceptable internal consistency [104]. The MIKAT will be adapted to diminish its focus on MI for substance use treatment.
- Perceptions of Evidence-Based Practices (EBPs) will be evaluated using 4 self-report measures:
  1. The Evidence-Based Practice Attitudes Scale-15 [105] is a 15-item assessment of providers’ attitudes toward EBPs, with 4 factors: Appeal, Requirements, Openness, and Divergence. Participants will also complete 2 assessments of their perceptions of how EBPs are viewed by their supervisor and at the agency.
  2. The Implementation Climate Scale [106], reliably assesses the extent to which a clinic fosters EBP implementation.
  3. The Implementation Leadership Scale [107] assesses leadership support for EBP implementation.
  4. TFA quantitative questionnaire [100]. Finally, we will assess the prospective acceptability of iMI4HIV using a tailored version of the generic TFA questionnaire. This questionnaire is composed of a 5-point Likert-type scale for each of the TFA components and 1 general question on acceptability. Findings from phase 1 will also identify other areas of inquiry for the baseline CASI.
- SPIs. We will use scheduling software for study participants to schedule their SPIs. At the specified time, the SPI actor will contact the participant by phone to conduct a 15-minute MI session focused on an HIV care scenario. The session will be audio-recorded and then rated using the Motivational Interviewing Treatment Integrity ratings (MITI 4.2) [108], a valid and reliable measure of MI skills [109]. MI skills will be assessed at baseline, postworkshop, post iMI4HIV, and 3 months later (for participants randomized into iMI4HIV).

#### MI Virtual Workshop

Synchronous VMI workshops will be conducted through Zoom (Zoom Video Communications, Inc). We expect 2 to 3 workshops with 10-15 CSS in each to facilitate individualized feedback during the workshops. Afterward, participants will receive a brief survey asking for feedback on the workshop.

#### Randomization

As shown in Figure 1 above, after completing the SPI-2, participants will be randomized into iMI4HIV or waitlist control at a 2:1 ratio. After completing the VMI workshop, iMI4HIV participants will be sent a link to set up their account and access iMI4HIV. They will be asked to complete the online training within 4 weeks. Participants will receive weekly reminders to complete the training within 4 weeks, at which time access to the program will end. Waitlist control individuals will not have access to iMI4HIV during the 4 weeks following the VMI workshop but will get access once their follow-up assessment and SPI-3 are completed.

#### Follow-up Assessment 1

Four to 5 weeks after randomization, participants in both arms will complete the first follow-up assessment, which forms the basis for comparing the 2 RCT arms and will consist of a CASI and SPI #3. Participants in the iMI4HIV arm will also take part in a QI. Participants in the iMI4HIV arm who did not complete the program will still be invited to complete the follow-up assessment (for which there is an incentive) so we can learn about obstacles to completing the program and obtain their recommendations.

- Follow-up CASI. This assessment will repeat those measures conducted at baseline but tailored to each study arm. For the intervention arm, the follow-up CASI will additionally include iMI4HIV-specific assessments: (1) TFA-based questions to assess acceptability of iMI4HIV, (2) obstacles to completing iMI4HIV, and (3) self-report of any additional MI training (eg, watching YouTube videos and reading MI articles or books) in which participants engaged. Findings from phase 1 will also identify other areas of inquiry for the follow-up CASI.
- SPI-3. After completing the follow-up CASI, participants in both arms will complete the third SPI as previously described, but using a different HIV-related patient scenario.
- QIs with CSS. Participants in the iMI4HIV arm will then have a QI to explore topics including (1) the TFA constructs, (2) iMI4HIV components deemed enjoyable, educational, and helpful, (3) acceptability of different gamification components, (4) facilitators and obstacles to completing iMI4HIV, (5) perceptions of current competency in MI, including strengths and weaknesses, (6) factors that have facilitated or hindered the use of MI in HIV care interactions, and (7) recommendations for further revisions of iMI4HIV. QIs are expected to be approximately 60 minutes in duration, and will be audio-recorded, transcribed, and verified for accuracy before being deidentified and uploaded to NVivo (Lumivero) for coding and analysis.

#### Follow-up Assessment 2

The second follow-up assessment will vary based on the study arm. Once participants in the control arm have completed their Follow-up Visit 1, they will be given access to iMI4HIV. They will be asked to complete the program within 4 weeks, at which point they will complete Follow-up Visit 2, consisting of (1) follow-up CASI assessment, (2) SPI-4, and (3) a QI. This follow-up CASI will be the same as that completed by participants in the active iMI4HIV arm at their first follow-up assessment. The SPI-4 will use a different scenario from those used in previous SPIs and will be conducted using the same procedures as the baseline SPIs. The QI will use the same as guide for the first follow-up assessment for those in the iMI4HIV arm; however, it will include additional questions exploring the impact of delayed access to iMI4HIV after completing the virtual workshop. Intervention arm participants will complete their Follow-up Visit 2 three months after their Follow-Up Visit 1. The assessment will repeat the CASI assessment from the first follow-up assessment and they will complete SPI-4. This will allow us to explore the degree to which gains in MI skills at SPI-3 are retained after 3 months.

### Data Analysis

#### Qualitative Data Analysis for QIs

Transcripts from the phase 2 QI audio-recordings will be reviewed for accuracy and uploaded into NVivo for management and analysis. Codebook development will begin when 8 transcripts are available. First-level codes will be guided by the TFA components, while second- and third-level codes will emerge from themes identified in the narratives. An initial set of codes will be generated independently by 2 research staff, compared, then synthesized to compile shared coding categories and subcategories, all with definitions, inclusion and exclusion criteria, and examples. As transcripts become available, coding will continue, refining definitions that will be used to process the remainder of the manuscripts. Coders will discuss discrepancies until they achieve 80% intercoder convergence. As per Patton [110], we will identify indigenous (ie, participant-generated) as well as analyst-constructed typologies. Once data are coded, analysis of coding reports will include categorization, abstraction, comparison, integration, iteration, and refutation of themes.

#### Integration of Quantitative and Qualitative Data

We will exploit the richness of our mixed methods approach, with quantitative and qualitative findings enriching each other. Quantitative assessments allow us to systematically explore variables of interest across all participants. Qualitative data will add nuance and new insights to quantitative findings. During data analysis, interpretation of quantitative results will be enriched by the summary of codes for specific components of iMI4HIV. In NVivo, we will categorize participants based on their responses in the quantitative assessment and use these categories to compare and contrast themes and concepts that emerge from the qualitative data.

#### Quantitative Data Analysis

##### Primary Outcomes

The primary outcomes of the pilot study will be based on the phase 2 RCT pilot, including (1) the percent of study participants that complete iMI4HIV within 4 weeks posttraining workshop (feasibility) and (2) the percentage of participants that respond Acceptable or Completely Acceptable to the question, “How acceptable was iMI4HIV to you?” from the TFA generic acceptability questionnaire (acceptability).

##### Exploratory Outcomes

We will also explore the following outcomes:

- MI skills acquisition. MITI-4.2 ratings for (1) Technical Component, (2) Relational Component, (3) Percent Complex Reflections, (4) Reflection:Question Ratio, (5) Total MI Adherent, and (6) Total MI Non-Adherent. Although the pilot RCT is not powered to detect differences between the intervention and control groups, we will compare these scores between the 2 groups for the SPI-3 assessment and within groups for each of the SPI assessments.
- Feasibility. Metadata from iMI4HIV, including mean (1) duration to complete specific iMI4HIV tasks, (2) number of times a lesson was completed prior to achieving a passing score, and (3) score for each task.
- Acceptability. Mean ratings on each of the generic TFA items (Affective Attitude, Burden, Intervention Cohesiveness, Self-efficacy, Ethicality, Perceived Effectiveness, and Opportunity Cost).

Data collected via assessments will be imported into IBM SPSS Statistics to calculate descriptive statistics for the sample. Estimation of the feasibility and acceptability outcomes (primary and exploratory) will be accomplished by generating the point estimate with its corresponding 95% CI. We will use a *t* test for continuous outcomes, and Fisher exact test for binary outcomes for the above comparisons. Adjustment for covariates, if necessary, with control baseline values, will be accomplished by multiple linear (continuous outcomes) or logistic (dichotomous outcomes) regression. While this study will not recruit a large enough sample to provide adequate power for assessing the efficacy of iMI4HIV, we will use data from MITI-4 ratings to explore specific changes in MI skills (listed in exploratory outcomes, above). To examine the efficacy of the addition of iMI4HIV on MI skills, the generalized linear model with identity and logit link function will estimate model parameters and their SEs for continuous and dichotomous variables, respectively. The generalized estimating equation method, with a robust SE estimator, will account for the within-subject correlation due to repeated measures from the same participant. Accompanying each of these tests will be point and interval estimates for the parameters of interest, such as group mean differences and odds ratios.

##### Power Considerations

There is some controversy regarding the use of pilot data to estimate an actual effect size [111]; clinically meaningful effect sizes should be decided based on extrinsic, clinical judgment grounds, and not based on pilot data which are too few, typically, to obtain reliable conclusions. We can use pilot data to rule out unusually large or small true effects through standard 95% CI procedures. We will confirm that extrinsic effect sizes are contained within our CIs from the pilot. We will examine the distribution of each variable and calculate summary statistics by intervention condition. We will estimate key intervention parameters with sample means and proportions together with 2-sided 95% CIs and test the primary null hypotheses at the traditional 2-sided level α=.05 (to simulate the subsequent RCT). For planning the RCT, we will also consider 1-sided 90% confidence limits for mission-critical design parameters such as SDs and reference group end point rates and proportions in the conservative direction. This is because we intend to plan the sample size for the RCT so that power will be “excellent” (at least 80% power) for the clinically relevant effect size to be specified if our estimate of SDs is at the 1-sided 90% confidence limit in the conservative direction. This strategy will make proper allowance for the limited sample size of the pilot study with its consequent uncertainties, and still yield appropriate sample sizes for the future RCT. We will examine the strength of association between our primary outcome and key variables, with the view toward seeing if stratifying on such factors (eg, age and gender) would substantially reduce variability and increase the power of a future RCT. “Mission-critical” parameters include proportions for dichotomous end points; means and SDs for continuous end points; and response profiles for longitudinal studies. Large sample sizes are not required to locate these parameters approximately, but adequately, for planning the subsequent trial, whereas testing the study hypothesis in the pilot will generally not have sufficient statistical power. Estimation of mission-critical design parameters with point and CI estimates will be very important; continuous outcome measures make it feasible to detect promising effects even in small samples. For the continuous measures, means and SDs will be estimated. For approximately normally distributed variables, an upper 1-sided 90% confidence limit for the population SD, *s_U_*, will be constructed from *s_U_* = *s* × [(n – 1)/*x*^2^_0.10,_*_n_*_–1_]^1/2^, where *s* is the sample SD and *x*^2^_0.10,_*_n_*_–1_ is the upper 10th percentile of the chi-square distribution with *n* – 1 degrees of freedom. For approximately log-normally distributed variables, a logarithmic transformation will be applied to achieve approximate symmetry and normality. For the dichotomous variable, exact binomial methods will be used.

### Ethical Considerations

The study was reviewed and approved by the Florida State University (FSU) Institutional Review Board (ID: STUDY00004477). This study does not assess health-related biomedical or behavioral outcomes; thus, it does not meet NIH criteria for a clinical trial and will not be registered in ClinicalTrials.gov.

All participants are provided with a written informed consent form to read before participating in the study. The consent form includes an overview of the study, study procedures, risks of participation, procedures to minimize risks of participation, a statement of voluntary participation, and contact information for the principal investigators, along with contact information for the FSU Institutional Review Board.

The results of the study may be published or presented, but no information that can identify individual participants will ever be provided or released in publications or presentations. To protect the privacy and confidentiality of participants, the study staff will keep any records containing identifying information in a locked cabinet accessible only to the research staff. The computer programs used in tablet-based interventions are compliant with US regulations on personal health information, as are the servers on which the data are stored. All data captured on the tablet, although it do not include identifying information, are encrypted in transit to maintain confidentiality. Any other study data will be stored on a secure computer that is protected by passwords. All records will be kept confidential. This is consistent with the guidelines of the US NIH. After removing all identifying information, the remaining deidentified information collected during the study could be used for future research studies or distributed to another investigator for future research studies without additional consent.

All participant incentives will be paid via gift cards. In phase 1, FG participants received US $75; agency leaders received US $100 for the interview; and iMI4HIV pilot participants will receive US $50 plus the opportunity to win a US $250 prize for completing the intervention within the 4-week allotted period. This incentive is provided to motivate the timely completion of the online training program among these participants to inform any final revisions that are necessary before the phase 2 RCT; main study outcomes are based on the phase 2 RCT results, which do not include this raffle incentive. Phase 2 RCT participants will receive up to US $205 for completing questionnaires (3 timepoints), 4 standard patient interactions, and a qualitative interview.

## Results

The study launched in February 2024. During the summer of 2024, we conducted 2 FGs (n=11) and QIs (n=8) with HIV Care Agency Leadership to inform the content and interactive components of the iMI4HIV. Preliminary findings from the FGs and QIs were shared with the CAB and expert consultants. In the Fall of 2024, we recorded a series of training videos focused on using MI in key HIV care interactions. Since then, we have been developing the content and layout of the training program. We expect to conduct the phase 1 pilot study in the Fall of 2025 and the pilot RCT during 2026.

## Discussion

### Overview

To address the need for online MI training geared specifically for the HIV care setting, this mixed methods study seeks to develop iMI4HIV, an interactive, gamified, online MI training program specifically tailored for CSS to improve their MI skills in HIV care communications. The goal is to train CSS to deliver MI competently so they can integrate MI into their health care communications with individuals seeking or receiving HIV-related services (status-neutral). If successful in building MI skills among CSS, iMI4HIV has the potential to shift how MI training is provided to CSS who work in HIV care settings. Furthermore, the program can be housed at FSU for use by AETCs throughout the country to provide additional MI training to their client agencies, used to train HIV treatment counselors in research studies, and provide training opportunities for other community-based agencies working to end the HIV Epidemic.

### Strengths

This proposed study has numerous strengths. First, contrary to the currently available simulations for MI training, iMI4HIV will offer an innovative, sequenced approach to comprehensive online MI training with a range of interactive and gamification components used in novel ways to build key MI technical skills. As such, it will also be the first to assess user acceptability, feasibility, and engagement of this new approach to online MI training. Second, at an expected 8-10 hours, iMI4HIV will be the first and most comprehensive interactive online MI training that addresses the full status-neutral HIV care cascade. The only existing interactive HIV-focused MI training is approximately 1 hour in duration and only addresses treatment adherence counseling for nurses. Third, this is the first online MI training study to systematically engage targeted end users throughout the development and testing process to obtain their feedback for revisions. Fourth, based on current scientific literature, this study will be among the first to conduct a rigorous assessment of efficacy (even within an underpowered RCT).

### Limitations

Although we anticipate high interest in MI training and iMI4HIV, recruitment and retention challenges can always arise and we consider these the main potential challenges to this study. Additionally, acceptability data (both quantitative and qualitative) are subject to social desirability. To minimize this risk, we will highlight for participants the importance of openly sharing their experiences and opinions, whether positive or negative.

### Conclusions

As designed, this study specifically responds to the Notice of Special Interest: Advancing Health Communication Research on HIV Prevention, Treatment and Cure (NOT-MH-21-105) announced by NIH, which includes an interest in studies that seek to “optimize effective communication and engagement practices between PLHIV and healthcare providers.” This R34 begins to address a critical need for novel approaches to train CSS in MI, a counseling or communication approach with proven efficacy in improving health care outcomes across the HIV care cascade. With its focus specifically on MI in HIV care interactions, iMI4HIV gives CSS in HIV care settings an invaluable opportunity to solidify MI skills gained during MI training workshops. Furthermore, as an asynchronous training program, iMI4HIV provides significant flexibility in when it can be completed, increasing accessibility to potential users. If findings on feasibility (≥70% of participants complete the iMI4HIV modules within 4 weeks) and acceptability (≥80% of participants rate iMI4HIV a “4” or “5” on the TFA overall acceptability rating) are favorable, we will submit a proposal for a fully powered RCT to assess the efficacy of iMI4HIV to achieve MI competence. In that study, we may even consider a study arm using an expanded version of iMI4HIV to allow for fully asynchronous MI training, which was cost-prohibitive in this study.

## Appendix

Multimedia Appendix 1 Peer-review report from HIBI - HIV/AIDS Intra- and Inter-personal Determinants and Behavioral Interventions Study Section, Risk, Prevention and Health Behavior Integrated Review Group (National Institutes of Health, USA).

## Abbreviations

AETC: AIDS Education and Training Center
CAB: Community Advisory Board
CASI: computer-assisted self-interview
CHW: community health worker
CSS: counseling support staff
EBP: evidence-based practice
FG: focus group
FSU: Florida State University
HCC: HIV Care Continuum
HIPAA: Health Insurance Portability and Accountability Act
iMI4HIV: internet-based motivational interviewing training program for HIV counseling support staff
MI: motivational interviewing
MIKAT: Motivational Interviewing Knowledge and Attitudes Test
MITI: Motivational Interviewing Treatment Integrity
NECA AETC: Northeast and Caribbean AIDS Education and Training Center
NIH: National Institutes of Health
QI: qualitative interview
RAD: Rapid Application Development
RCT: randomized controlled trial
SPI: Standard Patient Interaction
TFA: Theoretical Framework of Acceptability
UCRAD: User Centered Rapid Application Development
VMI: virtual motivational interviewing

## Data Availability

Data to be shared will be deposited with the digital repository of the Inter-university Consortium for Political and Social Research (ICPSR) to ensure that the research community has long-term access to the data. Once data have been deidentified and uploaded to a data repository, it will also be available on request from the investigators.
