# Supplementary material for: Interactive Internet-Based Motivational Interviewing Training for HIV Counseling Support Staff to Improve Health Communication in HIV Care Interactions: Protocol for Training Development and a Pilot Randomized Controlled Trial
Source: JMIR Res Protoc. 2026 Jan 7;15:e82241. doi: 10.2196/82241 (PMC12824569; doi:10.2196/82241)
Supplement: Multimedia Appendix 1 [file resprot_v15i1e82241_app1.pdf]

SUMMARY STATEMENT

(Privileged Communication)

PROGRAM CONTACT:
Gregory Greenwood
(240) 669-5532
gregory.greenwood@nih.gov

Release Date: 07/29/2023
Revised Date:

Application Number: 1R34MH133468-01A1
Formerly: 1R34MH133468-01

Principal Investigator

BALAN, IVAN C

Applicant Organization: FLORIDA STATE UNIVERSITY

Review Group: HIBI
HIV/AIDS Intra- and Inter-personal Determinants and Behavioral Interventions Study Section
AIDS - EXP. REV.

Meeting Date: 07/10/2023
Council: OCT 2023
Requested Start: 12/01/2023
Opportunity Number: PAR-23-060
PCC: 9A-ASPD

Project Title: Interactive Internet-based Motivational Interviewing Training for HIV Counseling Support Staff to Improve Health Communication in HIV Care Interactions
SRG Action: Impact Score:20 Percentile:5 +
Next Steps: Visit https://grants.nih.gov/grants/next_steps.htm
Human Subjects: 30-Human subjects involved - Certified, no SRG concerns
Animal Subjects: 10-No live vertebrate animals involved for competing appl.
Gender: 1A-Both genders, scientifically acceptable
Minority: 1A-Minorities and non-minorities, scientifically acceptable
Age: 3A-No children included, scientifically acceptable

Project Year	Direct Costs Requested	Estimated Total Cost
1	175,000	277,964
2	150,000	238,255
3	125,000	198,546
TOTAL	450,000	714,765

ADMINISTRATIVE BUDGET NOTE: The budget shown is the requested budget and has not been adjusted to reflect any recommendations made by reviewers. If an award is planned, the costs will be calculated by Institute grants management staff based on the recommendations outlined below in the COMMITTEE BUDGET RECOMMENDATIONS section.

1R34MH133468-01A1 Balan, Ivan

RESUME AND SUMMARY OF DISCUSSION: This application seeks to conduct a mixed-methods study to develop and pilot test iMI4HIV which is a post-workshop online training program for counseling support staff (CSS; e.g., HIV counselors, community health workers). The program seeks to train the CSS to competently deliver Motivational Interviewing (MI) to improve their health communication in status-neutral HIV care interactions. The iMI4HIV program will be developed using focus groups, community advisory board feedback, MI training experts, and HIV care agency leaders. Then the feasibility and acceptability of the iMI4HIV program will be evaluated in a pilot randomized controlled trial that compares Virtual MI training workshop + iMI4HIV to the virtual MI training workshop + waitlist control in 30 CSS. In addition, the preliminary efficacy and the obstacles and facilitators to iMI4HIV will be explored. This application replies to a NOSI (NOT-MH-21-105) that seeks studies that improve communication and engagement between people living with HIV and healthcare providers and the committee felt that this effort to improve the use of MI by CSS working in HIV care settings was responsive to the NOSI. MI has been shown to improve HIV care outcomes and, therefore, the proposed iMI4HIV program has the potential to have a significant public health impact. The study team is very strong and Dr. Balan has demonstrated expertise in MI. The applicant has been highly responsive to the concerns that were raised in the prior review of this application. Many areas were clarified and additional requested details were provided about the MI training program, the gamification components, and the waitlist control. In addition, the CSS that will receive the iMI4HIV program have been more broadly drawn and the HIV care agency leaders will now be interviewed and assessed as part of the study. As a result, the committee had only minor remaining concerns that did not reduce their overall very high enthusiasm for the potential impact of the proposed study.

DESCRIPTION (provided by applicant): The overall goal of the proposed study is to develop and pilot an interactive, gamified, online, training program in Motivational Interviewing (MI) specifically for HIV counseling support staff (CSS), which includes HIV counselors and community health workers, to improve health communication in status-neutral HIV care interactions. MI is an empathic, collaborative, counselling and communication approach that has demonstrated efficacy in improving outcomes across the HIV Care Continuum (HCC) in adults and adolescents. It is embedded in HIV care guidelines and training in MI is often provided to CSS by state and regional agencies. However, achieving competency in MI is not easy and the training typically provided to CSS is insufficient to achieve competence in MI. To ensure that MI retains its demonstrated efficacy in improving HCC outcomes, we must develop scalable MI training approaches that can both reach CSS working in HIV care settings and effectively increase their MI skills. To address this dual need, this study seeks to develop iMI4HIV, an interactive, gamified, online, MI training program specifically tailored for CSS in HIV care settings to improve their MI skills in HIV care communications. The goal is to train CSS to deliver MI competently so they can integrate MI into all of their healthcare communications with individuals receiving or seeking HIV related services (status-neutral). CSS will be able to access iMI4HIVs after completing a live virtual MI introductory workshop, providing additional skills training that specifically targets MI skills shown by research to affect behavior change outcomes. Thus, iMI4HIV will fill that critical need for additional post-workshop training that is rarely available to community providers. The Specific Aims of this study are to: 1) Develop iMI4HIV, an interactive, online, gamified MI training program specifically tailored towards CSS in HIV care settings to improve their MI skills with HIV care clients; 2) Conduct a pilot randomized controlled trial (RCT) of Virtual MI training workshop + iMI4HIV vs. Virtual MI training workshop + waitlist control (N=30 CSS) to: a) assess the feasibility and acceptability of iMI4HIV as measured by the percent of participants who complete iMI4HIV and retrospective acceptability ratings of iMI4HIV (Primary Aim), and b) explore preliminary findings on the effects of iMI4HIV on MI skills acquisition (Exploratory Aim); and 3) Explore experiences (including obstacles and facilitators) of completing iMI4HIV via in-depth interviews. As designed, this study specifically responds to the Notice

of Special Interest: Advancing Health Communication Research on HIV Prevention, Treatment and Cure (NOT-MH-21-105) announced by NIH, which includes an interest in studies that seek to “optimize effective communication and engagement practices between PLHIV and healthcare providers.” If successful in building CSS MI skills, iMI4HIV has the potential to shift how MI training is provided to CSS who work in HIV care settings and capitalize on the demonstrated efficacy of MI to improve HIV care outcomes.

PUBLIC HEALTH RELEVANCE: If successful in building MI skills among HIV counseling support staff, iMI4HIV has the potential to shift how MI training is provided to these critical providers of HIV care services. Furthermore, the program can be housed at FSU for use by AETCs throughout the country to provide additional MI training to their client agencies, used to train HIV treatment counselors in research studies, and provide training opportunities for other community-based agencies working to End the HIV Epidemic. Lastly, it can provide a training model that can be adapted to training providers working with other patient populations.

CRITIQUE 1

Significance: 3
Investigator(s): 1
Innovation: 4
Approach: 3
Environment: 2

Overall Impact: This R34 revision application seeks to develop and pilot an interactive training program in Motivational Interviewing for HIV counseling support staff. Innovation in the project is represented in the gamification aspect of the online training program coupled with a strong focus on assessment of MI competencies. The application is led by accomplished PI in the area of MI approaches in the context of HIV interventions, and the assembled team is highly qualified in all areas required to successfully accomplish the project aims, and the scientific environment further supports the likelihood of success. Further strengths include a strong measurement plan, community engagement in the planning of the intervention, and thoughtful consideration of elements that may support implementation of the program should it ultimately be shown to be effective. Although a limitation exists in the rationale for the waitlist control, the strengths of the proposal outweigh this concern, and development of the intervention is likely to yield important findings that will inform development of a future trial.

1. Significance:

Strengths

- Motivational interviewing (MI), when conducted by a skilled practitioner, has been found to contribute positively to a number of HIV-related outcomes.
- Traditional MI trainings are resource intensive and therefore can be challenging to implement widely. An effective online training in motivational training has potential application for application to diverse HIV patient populations and outcomes.

Weaknesses

- None noted by reviewer.

2. Investigator(s):

Strengths

- The PI has a strong history of research and practice experience in Motivational Interviewing
- All other needed areas of expertise are represented in the application.

Weaknesses

- None noted by reviewer.

3. Innovation:

Strengths

- Gamification, skills training, and other elements that focus on mechanisms of effective MI application in an online format represents an innovative approach to workforce development.

Weaknesses

- None noted by reviewer.

4. Approach:

Strengths

- A community advisory group will support development of the intervention.
- The three-month assessment will examine maintenance of acquired competencies in MI delivery.
- Inclusion of clear metrics to guide decision making regarding advancement to a clinical trial.

Weaknesses

- Greater justification is needed for the selection of the waitlist control group.
- If in-person training is the gold standard for MI, then some evidence that the online version performs as well as or better than that standard may ultimately be important to assess; consideration of the control group in a future confirmatory trial and how this would inform feasibility testing in this application is lacking.

5. Environment:

Strengths

- The Florida Center for Interactive Media will collaborate on development and programming of the training.
- Strong letters of support included from collaborators.
- All other resources required for successful completion of the aims are available.

Weaknesses

- None noted by reviewer.

Study Timeline:

Strengths

- The timeline is appropriate to the goals of the project.

Weaknesses

- None noted by reviewer.

Protections for Human Subjects:

Acceptable Risks and/or Adequate Protections

- Potential risks have been fully described and plans for protections are adequate.

Data and Safety Monitoring Plan (Applicable for Clinical Trials Only):

Not Applicable (No Clinical Trials)

Inclusion Plans:

- Sex/Gender: Distribution justified scientifically
- Race/Ethnicity: Distribution justified scientifically
- For NIH-Defined Phase III trials, Plans for valid design and analysis: Not applicable
- Inclusion/Exclusion Based on Age: Distribution justified scientifically
- Inclusion focuses on training of HIV counseling support staff who are typically 18 and older; no exclusions planned based on sex/gender or race/ethnicity.

Vertebrate Animals:

Not Applicable (No Vertebrate Animals)

Biohazards:

Not Applicable (No Biohazards)

Resource Sharing Plans:

Not Applicable (No Relevant Resources)

Budget and Period of Support:

Recommend as Requested

- No costs requested for data management and sharing.

CRITIQUE 2

Significance: 2
Investigator(s): 3
Innovation: 3
Approach: 2
Environment: 3

Overall Impact: The proposed intervention development and evaluation study is simple and responsive revision. It proposes developing a new mobile intervention to address a well-defined problem by leading

experts in the area of using MI to improve HIV outcomes. The approach is rigorous, uses best practices in the field and will be conducted in a manner that should allow the intervention to accomplish its goal. Very few weaknesses were noted and those that were do not detract from the study's potential long-term impact on the field.

1. Significance:

Strengths

- Ability to sustain and grow counselling staff's skill's in MI in low burden way is a significant barrier to wider implementation of MI to improve HIV outcomes; this study will offer one possible solution which should (if successful) have cascading impact on the field
- Clearly responds to NIH"s NOT-MH-21-105 on Increasing HIV Communication and by targeting those counselling staff members who interact the most with those with or at risk for HIV, the resulting intervention is likely to increase health behaviors in this and perhaps other populations
- Addition of the critical literature review of all MI technology interventions clearly defines the need for this combination of and proposed solution

Weaknesses

- None noted by reviewer.

2. Investigator(s):

Strengths

- PI Balan has extensive experiencing in conducting and training in motivational interviewing, leading large and small HIV research studies and in successfully and widely disseminating this work; this should allow the team to complete the proposed aims in the timeline specified

Weaknesses

- Unclear if anyone of the team has experience in building interventions to be delivered in mobile format, gamification, and evaluating those interventions; However, the team's consultation with the FSU media center tempers this weakness

3. Innovation:

Strengths

- The proposed intervention uses a number of novel approaches (gamification, sequenced modules) to help sustain and grow support staff's MI skills and confidence in order to improve HIV outcomes

Weaknesses

- Scientific approaches, concepts and instruments are all standard

4. Approach:

Strengths

- Well defined study goal with clear and justified boundaries; enhances likelihood of study success and subsequent impact on the wider HIV clinical field

- Partnering with AETC to help build and test the intervention will help it to be tailored to a population this is most likely to use it and thereby increase its scalability
- Employing the User Centered Rapid Application Development model will help the team efficiently and comprehensively employ best practices when creating their intervention
- Clear feasibility and acceptability outcomes that are likely to inform next iterations of the intervention as well as clear decisions about if to proceed with proposing an efficacy trial

Weaknesses

- Unclear why simulations take 12-14 months to create one simulation and the team anticipates they can create all of their videos in 6-12 months

5. Environment:

Strengths

- Florida State University College of Medicine, the FSU Center for Interactive Media and Albert Einstein College of Medicine have many resources that should enable the study team to complete its proposed aims

Weaknesses

- Exactly how the resources will support the proposed aims are not described

Study Timeline:

Strengths

- Discrete project will clear milestones and outputs; likely to be completed in the timeline with few anticipated challenges

Weaknesses

- None noted by reviewer.

Protections for Human Subjects:

Acceptable Risks and/or Adequate Protections

- The very few potential risks for being enrolled in this study were identified and addressed in the mitigation plans

Data and Safety Monitoring Plan (Applicable for Clinical Trials Only):

Not Applicable (No Clinical Trials)

Inclusion Plans:

- Sex/Gender: Distribution justified scientifically
- Race/Ethnicity: Distribution justified scientifically
- For NIH-Defined Phase III trials, Plans for valid design and analysis: Not applicable
- Inclusion/Exclusion Based on Age: Distribution justified scientifically

Vertebrate Animals:

Not Applicable (No Vertebrate Animals)

Biohazards:

Not Applicable (No Biohazards)

Resubmission:

- Generally a responsive application that addressed weaknesses noted previously including more detailing on the gamification components, adding more acceptability interview and targeting the intervention more broadly to the range of counselling staff

Applications from Foreign Organizations:

Not Applicable (No Foreign Organizations)

- FSU is the primary applicant (domestic)

Resource Sharing Plans:

Not Applicable (No Relevant Resources)

Budget and Period of Support:

Recommend as Requested

- Modular budget that is well justified, lean and no overlap is noted

CRITIQUE 3

Significance: 2
Investigator(s): 1
Innovation: 3
Approach: 3
Environment: 2

Overall Impact: This is a responsive resubmission R34 application from a highly qualified investigator team that proposes to develop and pilot test an online, interactive motivational training program for HIV counseling support staff (iMI4HIV). Focus groups will be conduced with staff and organization leaders and then pilot tested in an RCT (n=30) of staff. Strengths of the application include the study team, research environments, and the generally sound scientific approach that addresses key components of pilot intervention trials. Some details are lacking about feasibility assessments and measurement of fidelity to the iMI4HIV intervention, but these are more minor concerns that could be addressed. On balance, this is a strong application with moderate to high likelihood of making a significant public health impact.

1. Significance:

Strengths

- Lack of follow-up MI training often leads to deterioration of MI skills learned in training workshops. The proposed work addresses this training gap.

- The proposed iMI4HIV intervention addresses many of the limitations of existing online and simulated MI trainings.

Weaknesses

- None noted by reviewer.

2. Investigator(s):

Strengths

- The team possesses considerable expertise in MI interventions for persons living with and impacted by HIV.
- Drs. Balan and Kutner are both long-standing members of the MINT.
- The team has experience conducting pilot studies that form the basis for larger scale RCTs.

Weaknesses

- None noted by reviewer.

3. Innovation:

Strengths

- Offering sequenced training in a digital format to advance MI skill development is novel.

Weaknesses

- Online MI trainings are not particularly innovative.

4. Approach:

Strengths

- Designing an MI training that is suitable for a variety of learners enhances future dissemination potential.
- Inclusion of a Community Advisory Board to guide research activities is a strength.
- The User Centered Rapid Application Development process guides activities and provides a systematic approach to development of iMI4HIV.
- The analytic approach is sound and appropriate for a pilot trial.

Weaknesses

- The rationale for a waitlist control is still somewhat weak. The argument for iMI4HIV is that it addresses weaknesses of existing simulated trainings that may be used in HIV care settings. Why then not use an existing simulated training as the comparator?
- A plan for evaluating the fidelity to the training, iMI4HIV, is not presented.
- The application does not describe assessment of feasibility for other study activities (e.g., recruitment) that will be critical for a larger scale trial.
- There is come confusion about the inclusion of mental health providers. The research strategy states that master’s-level and licensed counselors will be included, while the recruitment and retention plan states mental health providers are excluded.

5. Environment:

Strengths

- FSU and Albert Einstein College of Medicine possess the resources needed to execute this study.
- The FSU Center for Interactive Media is critically important to the development of iMI4HIV.

Weaknesses

- None noted by reviewer.

Study Timeline:

Strengths

- Appropriate timeline for recruitment, data collection, intervention/training, and data analysis. All appears feasible in the timeline specified.

Weaknesses

- None noted by reviewer.

Protections for Human Subjects:

Acceptable Risks and/or Adequate Protections

- Minimal risk study with adequate protections. No health information is collected from subjects.

Data and Safety Monitoring Plan (Applicable for Clinical Trials Only):

Acceptable

- Detailed and appropriate

Inclusion Plans:

- Sex/Gender: Distribution justified scientifically
- Race/Ethnicity: Distribution justified scientifically
- For NIH-Defined Phase III trials, Plans for valid design and analysis: Not applicable
- Inclusion/Exclusion Based on Age: Distribution justified scientifically
- All ages over 18 included; all genders, likely equal numbers of men and women, some transgender; all races and ethnicities included, to resemble the population served by the CSS--greater than 80% minoritized race and/or ethnicity.

Vertebrate Animals:

Not Applicable (No Vertebrate Animals)

Biohazards:

Not Applicable (No Biohazards)

Resubmission:

- This resubmission is responsive to prior reviewer comments.

Resource Sharing Plans:

Acceptable

Budget and Period of Support:

Recommend as Requested

THE FOLLOWING SECTIONS WERE PREPARED BY THE SCIENTIFIC REVIEW OFFICER TO SUMMARIZE THE OUTCOME OF DISCUSSIONS OF THE REVIEW COMMITTEE, OR REVIEWERS' WRITTEN CRITIQUES, ON THE FOLLOWING ISSUES:

PROTECTION OF HUMAN SUBJECTS: ACCEPTABLE

INCLUSION OF WOMEN PLAN: ACCEPTABLE

INCLUSION OF MINORITIES PLAN: ACCEPTABLE

INCLUSION ACROSS THE LIFESPAN: ACCEPTABLE

COMMITTEE BUDGET RECOMMENDATIONS: The budget was recommended as requested.

Footnotes for 1R34MH133468-01A1; PI Name: Balan, Ivan C

+ Derived from the range of percentile values calculated for the study section that reviewed this application.

NIH has modified its policy regarding the receipt of resubmissions (amended applications).See Guide Notice NOT-OD-18-197 at https://grants.nih.gov/grants/guide/notice-files/NOT-OD-18-197.html. The impact/priority score is calculated after discussion of an application by averaging the overall scores (1-9) given by all voting reviewers on the committee and multiplying by 10. The criterion scores are submitted prior to the meeting by the individual reviewers assigned to an application, and are not discussed specifically at the review meeting or calculated into the overall impact score. Some applications also receive a percentile ranking. For details on the review process, see http://grants.nih.gov/grants/peer_review_process.htm#scoring.

MEETING ROSTER

HIV/AIDS Intra- and Inter-personal Determinants and Behavioral Interventions Study Section

Risk, Prevention and Health Behavior Integrated Review Group
CENTER FOR SCIENTIFIC REVIEW
HIBI
07/10/2023 - 07/11/2023

Notice of NIH Policy to All Applicants: Meeting rosters are provided for information purposes only. Applicant investigators and institutional officials must not communicate directly with study section members about an application before or after the review. Failure to observe this policy will create a serious breach of integrity in the peer review process, and may lead to actions outlined in NOT-OD-22-044 at https://grants.nih.gov/grants/guide/notice-files/NOT-OD-22-044.html, including removal of the application from immediate review.

CHAIRPERSON(S)

SAFREN, STEVEN A, PHD
PROFESSOR
DEPARTMENT OF PSYCHOLOGY
COLLEGE OF ARTS AND SCIENCES
UNIVERSITY OF MIAMI
CORAL GABLES, FL 33124

MEMBERS

COMULADA, W. SCOTT, DRPH
PROFESSOR
DEPARTMENT OF HEALTH POLICY AND MANAGEMENT
UNIVERSITY OF CALIFORNIA, LOS ANGELES
LOS ANGELES, CA 90024

COOK, ROBERT L, MD *
PROFESSOR
DEPARTMENT OF EPIDEMIOLOGY
COLLEGE OF MEDICINE
UNIVERSITY OF FLORIDA
GAINESVILLE, FL 32610

DODGE, BRIAN M, PHD
PROFESSOR
DEPARTMENT OF HEALTH PROMOTION SCIENCES
MEL AND ENID ZUCKERMAN COLLEGE OF PUBLIC HEALTH
UNIVERSITY OF ARIZONA
TUCSON, AZ 85724

GAMAREL, KRISTINE E, PHD
ASSOCIATE PROFESSOR
DEPARTMENT OF HEALTH BEHAVIOR
AND HEALTH EDUCATION
SCHOOL OF PUBLIC HEALTH
UNIVERSITY OF MICHIGAN
ANN ARBOR, MI 48109

HEADS, ANGELA, PHD
ASSOCIATE PROFESSOR
DEPARTMENT OF PSYCHIATRY AND BEHAVIORAL
SCIENCES
UNIVERSITY OF TEXAS HEALTH SCIENCES CENTER
HOUSTON, TX 77054

HECKMAN, TIMOTHY G, PHD *
PROFESSOR AND ASSOCIATE DEAN
DEPARTMENT OF HEALTH PROMOTION AND BEHAVIOR
COLLEGE OF PUBLIC HEALTH
UNIVERSITY OF GEORGIA
ATHENS, GA 30602

IWELUNMOR, JULIET, PHD
PROFESSOR
DEPARTMENT OF BEHAVIORAL SCIENCE AND
HEALTH EDUCATION
COLLEGE FOR PUBLIC HEALTH AND SOCIAL JUSTICE
ST. LOUIS UNIVERSITY
ST. LOUIS, MO 63104

LOVEJOY, TRAVIS IAN, PHD
ASSOCIATE PROFESSOR
DEPARTMENT OF PSYCHIATRY
SCHOOL OF MEDICINE
OREGON HEALTH AND SCIENCE UNIVERSITY
PORTLAND, OR 97239

MARHEFKA, STEPHANIE L, PHD
PROFESSOR
COLLEGE OF PUBLIC HEALTH
UNIVERSITY OF SOUTH FLORIDA
TAMPA, FL 33612

MILLAR, BRETT M, PHD *
ASSISTANT PROFESSOR
DEPARTMENT OF PSYCHIATRY
INSTITUTE FOR HEALTH, HEALTH CARE POLICY AND AGING
RUTGERS UNIVERSITY
NEW BRUNSWICK, NJ 08901

MONTGOMERY, ELIZABETH T, PHD *
SENIOR RESEARCH EPIDEMIOLOGIST
WOMEN'S GLOBAL HEALTH IMPERATIVE
RESEARCH TRIANGLE INSTITUTE
BERKELEY, CA 94704

MUESSIG, KATHRYN E, PHD *
PROFESSOR
DEPARTMENT OF NURSING
FLORIDA STATE UNIVERSITY
TALLAHASSEE, FL 32306

NEWCOMB, MICHAEL E, PHD
ASSOCIATE PROFESSOR
DEPARTMENT OF MEDICAL SOCIAL SCIENCES
FEINBERG SCHOOL OF MEDICINE
NORTHWESTERN UNIVERSITY
CHICAGO, IL 60611

PATEL, VIRAJ V, MD, MPH
ASSOCIATE PROFESSOR
DEPARTMENT OF MEDICINE
ALBERT EINSTEIN COLLEGE OF MEDICINE
BRONX, NY 10467

RITCHWOOD, TIARNEY D, PHD *
ASSOCIATE PROFESSOR
DIVISION OF SOCIAL SCIENCES AND HEALTH POLICY
DEPARTMENT OF PUBLIC HEALTH SCIENCES
WAKE FOREST UNIVERSITY SCHOOL OF MEDICINE
WINSTON-SALEM, NC 27109

SABIN, LORA L, PHD
ASSOCIATE PROFESSOR
DEPARTMENT OF GLOBAL HEALTH
BOSTON UNIVERSITY SCHOOL OF PUBLIC HEALTH
BOSTON, MA 02118

SANTOS, GLENN-MILO, PHD
PROFESSOR
DEPARTMENT OF COMMUNITY HEALTH SYSTEMS
SCHOOL OF NURSING
UNIVERSITY OF CALIFORNIA SAN FRANCISCO
SAN FRANCISCO, CA 94143

SCHRIMSHAW, ERIC W, PHD *
PROFESSOR
DEPARTMENT OF POPULATION HEALTH SCIENCES
SCHOOL OF MEDICINE
UNIVERSITY OF CENTRAL FLORIDA
ORLANDO, FL 32827

SIEGLER, AARON J, PHD *
ASSOCIATE PROFESSOR
DEPARTMENT OF EPIDEMIOLOGY
EMORY UNIVERSITY
ATLANTA, GA 30322

STEWART, JENELL, DO *
ASSISTANT PROFESSOR
DEPARTMENT OF INFECTIOUS DISEASE
HENNEPIN HEALTHCARE RESEARCH INSTITUTE
MINNEAPOLIS, MN 55415

SUN, SHUFANG, PHD *
ASSISTANT PROFESSOR
DEPARTMENT OF BEHAVIORAL AND SOCIAL SCIENCES
BROWN UNIVERSITY
PROVIDENCE, RI 02912

TANNER, AMANDA E, PHD *
PROFESSOR
DEPARTMENT OF PUBLIC HEALTH EDUCATION
SCHOOL OF HEALTH AND HUMAN SCIENCES
UNIVERSITY OF NORTH CAROLINA GREENSBORO
GREENSBORO, NC 27402

THAMES, APRIL D, PHD
PROFESSOR
DEPARTMENT OF PSYCHIATRY AND BEHAVIORAL SCIENCES
UNIVERSITY OF CALIFORNIA, LOS ANGELES
LOS ANGELES, CA 90095

TIEU, HONG VAN N, MD *
HEAD
LABORATORY OF INFECTIOUS DISEASE PREVENTION
LINDSLEY F KIMBALL RESEARCH INSTITUTE
NEW YORK BLOOD CENTER
NEW YORK, NY 10065

WEBEL, ALLISON R, PHD
PROFESSOR
SCHOOL OF NURSING
UNIVERSITY OF WASHINGTON
SEATTLE, WA 98195

WILSON, TRACEY E, PHD
VICE DEAN FOR FACULTY AFFAIRS AND RESEARCH
DEPARTMENT OF COMMUNITY HEALTH SCIENCES
SCHOOL OF PUBLIC HEALTH
SUNY DOWNSTATE HEALTH SCIENCES UNIVERSITY
BROOKLYN, NY 11203

WILTON, LEO, PHD
PROFESSOR
DEPARTMENT OF HUMAN DEVELOPMENT
COLLEGE OF COMMUNITY AND PUBLIC AFFAIRS
BINGHAMTON UNIVERSITY
BINGHAMTON, NY 13902

WINDSOR, LILIANE C, PHD
PROFESSOR
SCHOOL OF SOCIAL WORK
THE UNIVERSITY OF ILLINOIS AT URBANA-CHAMPAIGN
URBANA, IL 61801

SCIENTIFIC REVIEW OFFICER

RUBERT, MARK P, PHD
SCIENTIFIC REVIEW OFFICER
CENTER FOR SCIENTIFIC REVIEW
NATIONAL INSTITUTES OF HEALTH
BETHESDA, MD 20892

EXTRAMURAL SUPPORT ASSISTANT

MACPHERSON, SETH LOPAKI
EXTRAMURAL SUPPORT ASSISTANT
CENTER FOR SCIENTIFIC REVIEW
NATIONAL INSTITUTES OF HEALTH
BETHESDA, MD 20892

* Temporary Member. For grant applications, temporary members may participate in the entire meeting or may review only selected applications as needed.

Consultants are required to absent themselves from the room during the review of any application if their presence would constitute or appear to constitute a conflict of interest.
